# Nucleic Acids as Biotools at the Interface between Chemistry and Nanomedicine in the COVID-19 Era

**DOI:** 10.3390/ijms23084359

**Published:** 2022-04-14

**Authors:** Nicola Borbone, Ilaria Piccialli, Andrea Patrizia Falanga, Vincenzo Piccialli, Giovanni N. Roviello, Giorgia Oliviero

**Affiliations:** 1 Department of Pharmacy, University of Naples Federico II, Via Domenico Montesano 49, 80131 Naples, Italy; nicola.borbone@unina.it (N.B.); andreapatrizia.falanga@unina.it (A.P.F.); 2 Division of Pharmacology, Department of Neuroscience, Reproductive and Odontostomatological Sciences, University of Naples Federico II, Via Sergio Pansini 5, 80131 Naples, Italy; ilaria.piccialli@unina.it; 3 Department of Chemical Sciences, University of Naples Federico II, Via Cintia 26, 80126 Naples, Italy; vinpicci@unina.it; 4 Institute of Biostructures and Bioimaging, Italian National Council for Research (IBB-CNR), Area di Ricerca Site and Headquarters, Via Pietro Castellino 111, 80131 Naples, Italy; 5 Department of Molecular Medicine and Medical Biotechnologies, University of Naples Federico II, Via Sergio Pansini 5, 80131 Naples, Italy; giorgia.oliviero@unina.it

**Keywords:** DNA, RNA, oligonucleotides, nucleic acid analogs, COVID-19, antisense, mRNA vaccines, antigene, nanomedicine

## Abstract

The recent development of mRNA vaccines against the SARS-CoV-2 infection has turned the spotlight on the potential of nucleic acids as innovative prophylactic agents and as diagnostic and therapeutic tools. Until now, their use has been severely limited by their reduced half-life in the biological environment and the difficulties related to their transport to target cells. These limiting aspects can now be overcome by resorting to chemical modifications in the drug and using appropriate nanocarriers, respectively. Oligonucleotides can interact with complementary sequences of nucleic acid targets, forming stable complexes and determining their loss of function. An alternative strategy uses nucleic acid aptamers that, like the antibodies, bind to specific proteins to modulate their activity. In this review, the authors will examine the recent literature on nucleic acids-based strategies in the COVID-19 era, focusing the attention on their applications for the prophylaxis of COVID-19, but also on antisense- and aptamer-based strategies directed to the diagnosis and therapy of the coronavirus pandemic.

## 1. Introduction

**Figure 1:**
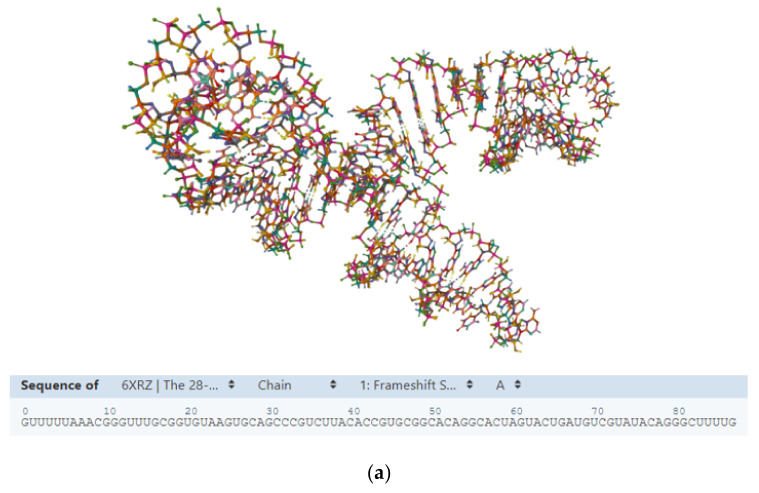

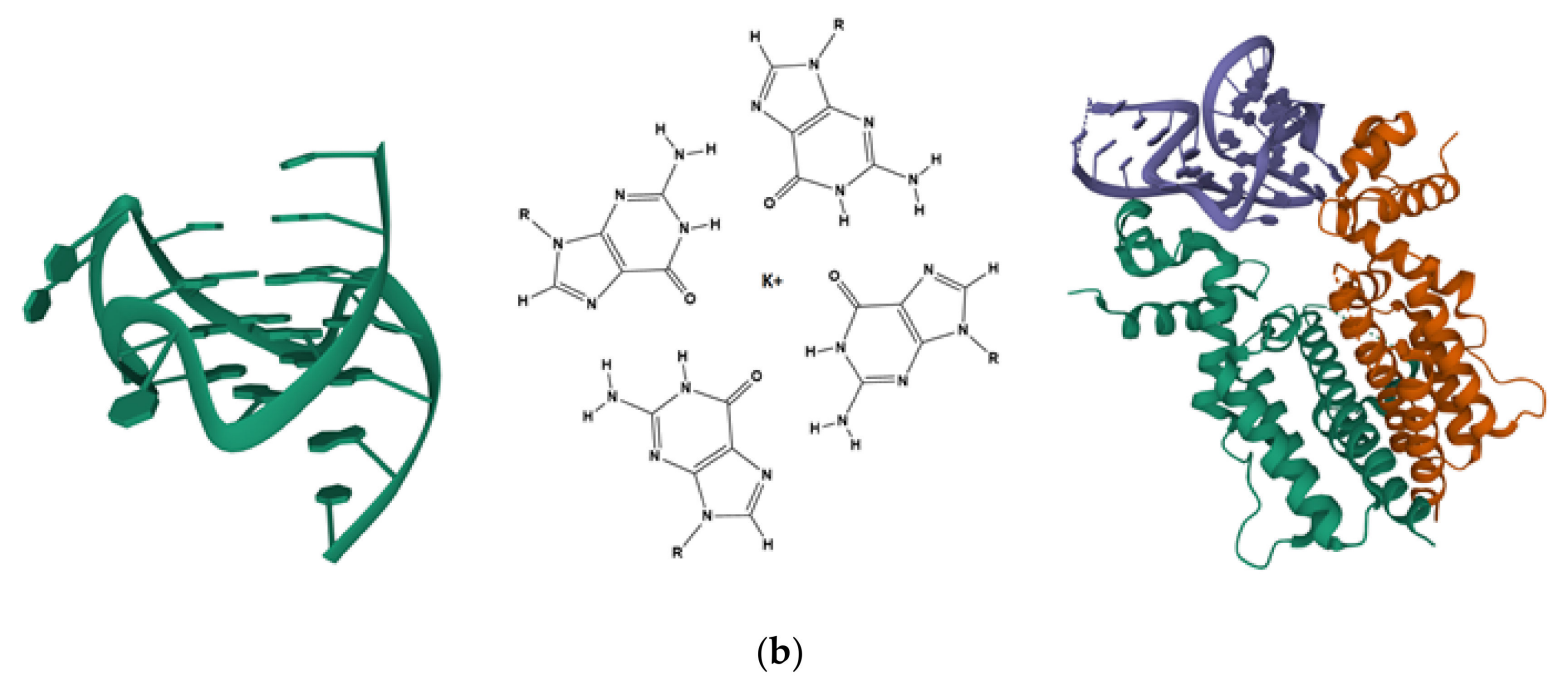
(**a**) The 28-kDa highly conserved FSE (frameshift stimulation element, PDB ID: 6XRZ https://www.rcsb.org/3d-view/6XRZ/1 accessed on 4 February 2022) of the SARS-CoV-2 genome is an example of a potential candidate for targeting by small molecules and oligonucleotides as it is required for the balanced expression of SARS-CoV-2 proteins [73]. (**b**) An example of quadruple helical DNA (a monomeric parallel-stranded quadruplex in human VEGF promoter; PDB ID: 2M27 https://www.rcsb.org/3d-view/2M27/0 accessed on 1 April 2022; **left**) with a schematic representation of a G4 quartet (**middle**); and a complex between a nucleic acid aptamer (RNA-aptamer K1; purple) with its protein target (Tetracycline repressor protein; PDB ID: 6SY4 https://www.rcsb.org/3d-sequence/6SY4?assemblyId=1 accessed on 1 April 2022; **right**).

The COVID-19 (coronavirus disease 19) pandemic, caused by severe acute respiratory syndrome coronavirus 2 (SARS-CoV-2), is causing enormous difficulties around the globe from both a sanitary and socioeconomic perspective [1,2,3,4,5,6,7,8,9,10]. Currently, the world is waiting for effective benefits following the mass vaccination campaign conducted in some parts of the globe using the developed anti-COVID-19 vaccines. However, despite the mass immunization campaign [11,12] and the efforts of pharmaceutical companies and the scientific community to devise effective therapies via new drug development [13,14], drug repurposing [15,16], herbal medicine [17,18,19,20,21] and other recently proposed approaches [22,23,24,25,26], SARS-CoV-2 and other human coronaviruses [27,28] remain a major global issue due to their mutations, leaving our future unclear. The recent development of DNA- and mRNA-carrying vaccines [29,30,31,32] able to elicit antibody production against the SARS-CoV-2 infection has recently recalled enormous attention to the potential uses of nucleic acids and their analogs as innovative biomedical tools. The oligonucleotide biotechnological use has been severely limited by their reduced half-life in the biological environment and the difficulties related to their delivery to target cells. Thus, some potent nucleic acid analogs (NAA) [33,34,35,36,37,38,39,40,41,42,43] are currently being utilized in anti-COVID-19 strategies because they allow one to overcome some of these limiting aspects by resorting to chemical modifications in the oligonucleotide and by using appropriate nanocarriers [44,45]. Like natural nucleic acids, NAAs can interact with complementary sequences of nucleic acid targets, forming stable complexes [46,47] and determining their loss of function [48]. For example, the binding of a peptide nucleic acid (PNA), locked nucleic acid (LNA), morpholino (PMO), or another synthetic analog [49] to the complementary nucleic acid target may determine (i) the inhibition of mRNA translation to the corresponding protein (antisense strategy), (ii) the blocking of gene transcription via specific binding with gene promoters (antigene strategy), and (iii) numerous biomolecular events exploited in several diagnostic applications [49]. An alternative strategy uses nucleic acids aptamers that, like the antibodies, bind to specific proteins to modulate their activity [50,51,52,53,54,55,56,57,58]. In this regard, i-motif [59,60] and G-quadruplex forming oligonucleotides [61,62,63] are particularly relevant as their specific role as aptamers was explored in vitro and, in some cases, find applications in biomedical strategies [64,65,66,67,68,69]. Drug discovery campaigns against COVID-19 are targeting not only viral proteins (such as M^pro^ main protease [70,71]) but also the viral RNA genome [72], with particular attention paid to highly conserved and expression-relevant tracts [73] (Figure 1).

This review will examine the recent literature on nucleic acid-based technologies in the COVID-19 era. We will focus on the main prophylactic, therapeutic and diagnostic DNA- and RNA-based tools currently under examination or in use in the fight against SARS-CoV-2.

## 2. DNA and RNA-Containing Vaccines in the Fight against COVID-19

Antiviral vaccines can generally be classified into three categories: (live-attenuated or inactivated) virus-based, protein-based, and nucleic acid-based [74]. The first two approaches have been the conventional methods that rely on unharmful forms of the virus or proteins directly delivered as immunogens to induce the immune response in the host. On the other hand, nucleic acid-based vaccines are delivered via nucleic acid vectors to host cells, where DNA or RNA genes will be expressed in the host to produce corresponding antigens that activate the adaptive and humoral immune response [74]. All three strategies have been explored for COVID-19, but currently (as of 4 February 2022), only gene vaccines are available in Western countries, even though the protein-based NVX-CoV2373 vaccine developed by the American Novavax should become available soon [75,76], and the inactivated whole virus vaccine VLA2001 from the French Valneva could be approved in the upcoming months [76]. One of the advantages of nucleic-acid-based vaccines is the easiness and relatively high rapidity of their manufacturing [74]. They can immediately be synthesized when the immunogen sequence is made available, and the process can be easily scalable. With respect to DNA-based vaccines, an mRNA vaccine expresses the antigen protein directly via translation from the mRNA after its transfection [74]. These vaccines are believed to possess higher biosafety than DNA-based vaccines because the mRNA is less likely integrated into the genome than a DNA-based vaccine, as the translation of the antigens in the case of mRNA vaccines takes place in the cytoplasm and not in the nucleus, where the DNA vaccines start to work [77]. However, several studies suggest that the risk of genomic integration, even if diminished compared to DNA vaccines, also remains for those based on mRNA, considering that eukaryotic cells may exert, to some extent, a reverse transcription activity [78,79,80] that could produce DNA theoretically starting from the vaccine-delivered mRNAs [81,82]. An advantage of nucleic acid-based vaccines over protein-based vaccines is that they may lead to antigens better mimicking the viral protein structure, including the post-translational modifications. In fact, while protein-based vaccines are often produced from bacteria, mRNA vaccines are translated by the host translation machinery. Nonetheless, the novel NVX-CoV2373 vaccine was developed using insect cells that can perform most of the desired post-translational modifications. As for the storage and transportation, DNA and mRNA vaccines must be stored and transported at low or ultra-low temperatures [83], whereas inactivated virus- and protein-based vaccines [84] require less stringent conditions. However, innovative lipid nanoparticle technologies are significantly improving the stability characteristics of mRNA vaccines that may require less stringent conditions [83].

From a molecular perspective, one of the most reliable strategies to fight SARS-CoV-2 consists of the administration of the nucleic information (DNA or RNA) that the host cellular machinery uses for the production of SARS-CoV-2 spike (S) protein, which has been generally used as the antigen of DNA vaccines used against COVID-19 (Table 1). Once produced in human cells, S protein may provoke the immune system to respond with cellular and humoral defenses, retaining the information in memory immune cells. In this way, the organism is prepared to counteract virus infection in the case of subsequent exposure to SARS-CoV-2. Generally, these prophylactic agents are administered in a two-dose immunization scheme, with both injections being administered intramuscularly, often within a three-week interval. Interestingly, the nucleic acid-based vaccination is currently the most exploited anti-COVID-19 prophylaxis in the Western world, and here below, we will give some details on the currently most in-vogue vaccines belonging to this category of vaccines.

### 2.1. DNA-Carrying COVID-19 Vaccines

They are often classified as “virally vectored vaccines” or “adenovirus vector vaccines” being based on vaccine vectors, but they should be more correctly denominated as adenovirus vector-based DNA vaccines to underline the nature of their cargo [76]. The most widely utilized in Western countries, i.e., the ChAdOx1-S/AZD1222-Spike [85] vaccine developed by the University of Oxford in collaboration with AstraZeneca (Cambridge, UK) pharmaceutical company (Table 1), used a Chimpanzee non-replicating viral vector that contains synthetic DNA encoding the S protein of SARS-CoV-2. Thus, the ChAdOx1-S/AZD1222 expresses the S protein gene, which instructs the human cells to produce the protein, allowing the body to generate an effective immune response. Clinical trials showed efficacy in participants who received two doses of the vaccine irrespective of the interval between the doses of about 63.1%, based on a median follow-up of 80 days or higher when this interval was longer [86]. The vaccine was manufactured by SK Bioscience Co., Ltd. (Pangyo-ro, Korea), under the name ChAdOx1-S, and the Serum Institute of India (Pune, India) named COVISHIELD [87]. Even though it is less common than the ChAdOx1-S/AZD1222, the Ad26.CoV2.S vaccine developed by Janssen Pharmaceuticals Johnson & Johnson (Beerse, Belgium) is worth mentioning. Ad26.CoV2.S is a non-replicating viral vector vaccine consisting of a human adenovirus vector, with a DNA genome, into which has been inserted the gene that encodes the S protein of SARS-CoV-2. The efficacy was 66.0% in phase-3 clinical trials (Table 1) [88].

### 2.2. RNA-Carrying COVID-19 Vaccines (mRNA Vaccines)

**Figure 2:**
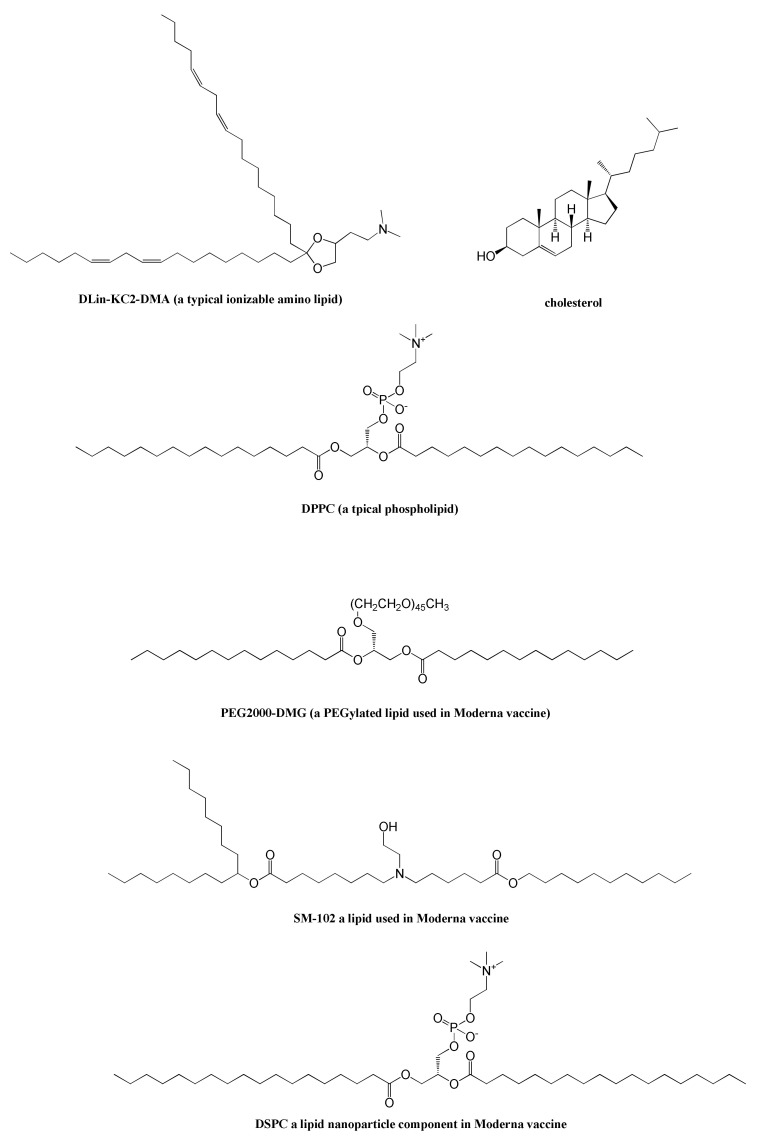
Chemical representations of some typical components of lipid nanoparticles as well as specific components of the Moderna vaccine. DPPC: dipalmitoylphosphatidylcholine; DSPC: distearoylphosphatidylcholine.

The most currently used vaccine type in Europe and North America, these vaccines exploit mRNAs to instruct human cells to produce the S protein [74]. Since mRNA vaccines do not need to reach the cell nucleus like the DNA-based ones, they are of higher practical significance. Although RNA is known to be a relatively unstable nucleic acid, novel vaccine nanotechnologies were developed to improve both mRNA stability and S protein translation efficiency, with consequent enhanced immune responses by the host cell. More in detail, lipid-nanoparticle encapsulation [89,90,91,92] of the vaccine is a strategy often used to optimize the delivery of the mRNAs for intradermal or intramuscular administration [93]. The leading exponents of this family of anti-COVID-19 prophylactic agents are BNT162/Comirnaty and mRNA-1273 (Table 1) [94]. The former is a lipid-nanoparticle encapsulated mRNA-based vaccine developed by BioNTech (Mainz, Germany) and Pfizer (New York, NY, USA), partnered with Fosun Pharma (Shangai, China), which encodes the RBD (receptor-binding domain) domain of the SARS-CoV-2 S protein. BNT162, loaded into a patented lipid-nanoparticle composed of ionizable amino lipid, phospholipid, cholesterol, and a PEGylated lipid (at a ratio of 50:10:38.5:1.5 mol/mol, Figure 2) [95], uses a modified 4284 nucleotides long mRNA [96] and includes the T4 fibrin-derived trimerization domain, which serves to enhance the immune response [88]. On the other hand, mRNA-1273, developed by Moderna (Cambridge, MA, USA) in collaboration with the American National Institute of Allergy and Infectious Diseases (NIAID, Bethesda, MD, USA), is a vaccine based on a 4004 nucleotides long mRNA [97] expressing the full-length prefusion stabilized S protein of SARS-CoV-2. In mRNA-1273, the mRNA strand is encapsulated by two proprietary cationic lipidic nanoparticles (WO2017070626 and WO2018115527) whose composition was described as SM-102, polyethylene glycol-2000-dimyristoyl glycerol (PEG2000-DMG), cholesterol, and 1,2-distearoyl-sn-glycero-3-phosphocholine (DSPC, Figure 2) [95].

As for dosages/schedule, routes of administration, and efficacies, two intramuscular doses are needed for both mRNA vaccines, 21 days apart (30 μg per dose) for BNT162/Comirnaty and 28 days apart (100 μg per dose) in the case of mRNA-1273. The confirmed efficacy of the BNT162/Comirnaty vaccine is 95.0% (measured starting from seven days after the second dose), while that of mRNA-1273 is 94.1% (measured starting from two weeks after the second dose) (Table 1) [98].

### 2.3. Chemical and Nanotechnological Optimization of mRNA Vaccine Design

Though exempt from any risks of genomic integration [77], the typical vaccine development using viruses or protein-based systems involves time-consuming steps that appear impractical for responding rapidly to any pandemic caused by newly emerging pathogens. As explained above, nucleic acid-based vaccines possess distinctive advantages of rapid development and versatility that led to the development of multiple COVID-19 DNA and mRNA vaccines. Among these, mRNA vaccines seem to have higher protective efficacy than DNA vaccines, but special strategies are needed to guarantee their safety, stability, and consequent efficacy due to some intrinsic RNA molecular features. In fact, RNA is particularly prone to enzymatic degradation by the RNases present in the plasma and serum. Moreover, RNA molecules in mRNA vaccines, being exogenous molecules, are seen by the human cellular machinery as an immunological mimic of viral infection, which provokes an immediate immune response by host cells. Hence, the importance of nanotechnologies aimed at maximizing the stability of RNA in mRNA vaccines and limiting the innate immune response in the host [99,100].

**Figure 3:**
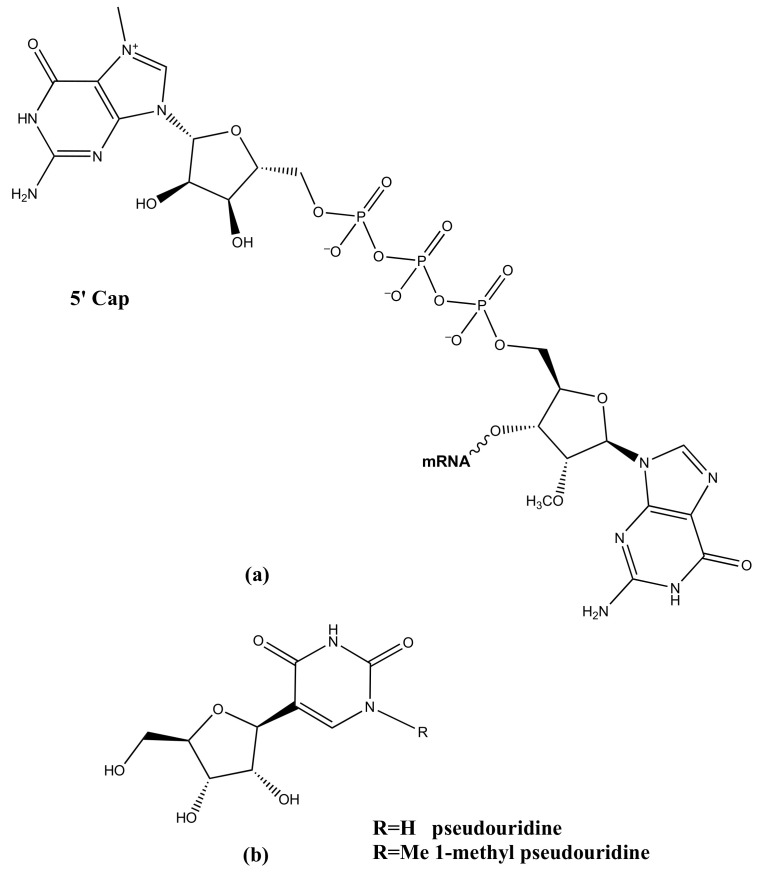
Schematic representation of some typical modifications of synthetic mRNAs contained in the COVID-19 vaccines: (**a**) 5′ capping via cap1 structure (m7GpppNm); (**b**) uridines are replaced with pseudouridine or 1-methyl pseudouridine units.

Endogenously, mRNAs undergo post-transcriptional modifications, such as 5′-capping [101] and polyadenylation [102,103], needed for mRNA stabilization (protecting mRNA from exonuclease activity) and efficient translation (facilitating pre-mRNA splicing and serving as the binding site for the translation initiation complex). 5′-capping includes the addition of 7-methylguanosine (m7G) at the 5′ end of the first ribonucleotide of mRNA molecules via a 5′ to 5′ linkage and the methylation of the 2′-OH of the ribose moiety of the same ribonucleotide to form m7GpppNm (Figure 3a). Interestingly, the host can discriminate between the self versus the exogenous mRNA thanks to the presence of the 5′-cap. The last observation explains why adding an m7GpppNm cap to the 5′-end of mRNA vaccines’ RNA strand is a highly desirable mRNA modification [101]. The polyadenylation (i.e., the addition of a poly rA tail at the 3′-OH of pre-mRNA) is another factor that stabilizes mRNA and promotes protein translation [102,103], as the length of the poly(A) tail is closely associated with the translation efficiency. However, the information on the nature of the poly(A) signal sequence was reported only in the case of the BNT162/Comirnaty vaccine [poly rA tail: A30(GCATATGACT)A70], whereas it remains proprietary and undisclosed for the Moderna’s counterpart [96].

Nucleoside modifications and, especially, the incorporation of pseudouridine (Figure 3b) into mRNA molecules may suppress the immune response by evading the activation of TLR-3, -7, and -8. Consequently, in the BNT162/Comirnaty vaccine, uridine residues were replaced by 1-methyl pseudouridine modification to reduce the innate immune response and enhance the stability of the exogenous mRNA [104].

Many liposome-based transfection reagents based on cationic lipids have been formulated to improve the transfection efficiency of mRNAs, which is generally low in the case of naked oligoribonucleotides [105]. Generally, the lipid components of mRNA vaccines are proprietary and include different cationic polypeptides, positively charged lipids, polymers, dendrimers, or micelles [106]. Lipid nanoparticles encapsulate their mRNA cargo into the stable lipid bilayer, which is internalized by recipient cells via endocytosis. More in detail, after the injection, the mRNA-lipid nanoparticle complexes enter muscle cells via endocytosis pathways, and then the translation of the mRNA leads to translates forming a metastable trimeric prefusion spike protein. Blood vessels adjacent to the muscles are then believed to recruit infiltrating antigen-presenting cells [107]. 

### 2.4. Nanotechnologies in the Development of Potential COVID-19 Vaccines

**Figure 4:**
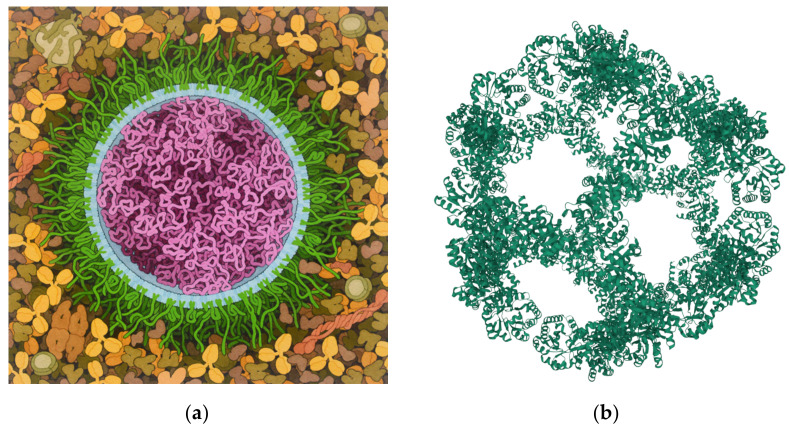
Structures of nanoparticles used for COVID-19 vaccine candidates: (**a**) mRNA vaccines realized for the COVID-19 pandemic are composed of long strands of RNAs (magenta) that encode the SARS-CoV-2 S protein enclosed in lipids (cyan), connected with lipids conjugated to polyethylene glycol (PEG) chains (green), that deliver the RNA cargo into recipient cells. In the idealized illustration by David S. Goodsell, RCSB Protein Data Bank [113], the lipids are arranged in a simplified model of a circular bilayer surrounding the mRNAs, and the PEG chains are endowed with both folded and extended conformations. Note how the real structure may be less regular, as suggested in the literature [110]. (**b**) An example of protein nanoparticle-based vaccine proposed against SARS-CoV-2. It displays the S glycoprotein receptor-binding domain (RBD) on a synthetic virus-like particle platform, SpyCatcher003-mi3, and uses SpyTag/SpyCatcher technology [112] (https://www.rcsb.org/structure/7B3Y, accessed on 4 February 2022).

Coronaviruses are nanoscale biostructures against which nanotechnology can be exploited to realize vaccines and immune engineering applications [108]. Live attenuated and inactivated vaccines, viral vectors, and mRNA-lipid nanoparticles [109,110] (Figure 4a) constitute examples of nano-biotechnological products so efficient against SARS-CoV-2. The use of nanomaterials as carriers of antigens or prophylactic mRNAs and DNAs is a recent biotechnological approach that proved successful in COVID-19 vaccine design technology. In a nanotechnological vaccine, antigens and nanoparticles mutually interact by adsorption, entrapment, and conjugation. As for the materials constituting the nanoparticles, liposomes, nanopolymers, quantum dots, and inorganic nanoparticles are conventional vehicles for subunit vaccines and nucleic acids [111]. Several types of lipids are also used, including cholesterol and ionizable lipids, which, being cationic, interact with the negatively charged RNAs.

Moreover, the conjugation of lipids to polyethylene glycol chains (Figure 4a, green) proved effective in shielding the mRNA cargo from the host immune system, thus prolonging a vaccine’s lifetime following intramuscular injection. Protein nanoparticles were also proposed in the realization of vaccines against SARS-CoV-2 (Figure 4b) [112]. The vaccine strategy, in this case, was based on the display of the S protein receptor-binding domain (RBD) on a synthetic virus-like particle platform, SpyCatcher003-mi3, using SpyTag/SpyCatcher technology [112].

## 3. DNA and RNA Targeting in the COVID-19 Era

### 3.1. RNA Targeting in the Diagnostics of COVID-19

Rapid screening of infected individuals from a large population is important in epidemiology, especially in controlling the spread of COVID-19 infections [114]. The reverse transcription polymerase chain reaction (RT-PCR) assay is the diagnostic standard for COVID-19. In contrast, rapid antigen tests based on lateral immunochromatography and using different matrices, including direct culture supernatants and dry swabs [115], could be used as point-of-care detection of SARS-CoV-2 antigens with several advantages over the RT-PCR assays including shorter turn-around times and lower costs [114]. However, their sensitivity in detecting the SARS-CoV-2 virus remains lower than RT-PCR (0.68 compared to RT-PCR), especially in low viral loads. Even though RT-PCR tests remain the gold standard for population-wide screening of COVID-19 and other epidemics, significant limitations prevent the large scale application of this technology, which include the significantly higher costs and longer turnaround times due to time-consuming nucleic acid extraction and amplification steps, and the required equipment for testing [114]. An RNA targeting strategy was thus ideated based on a direct nucleic acid assay that used a graphene field-effect transistor (g-FET) and Y-shaped DNA dual probes [116]. The method relied on Y-dual probes modified on g-FET simultaneously targeting nucleocapsid (N) and viral replicate polyprotein open reading frame (ORF1ab) genes of the SARS-CoV-2 RNA genome. Interestingly, the assay was associated with a high recognition ratio and a limit of detection one-two order of magnitude lower than other nucleic acid assays (0.03 copy μL^−1^). Another advantage of this RNA-targeting DNA-based assay was the rapidity of the nucleic acid testing (~1 min) compared to the longer times (up to 4 h) required by the other nucleic acid tests, along with the ultrasensitivity, easiness in operating features as well as capability in pooled testing [116].

### 3.2. COVID-19 Antisense Strategies

**Figure 5:**
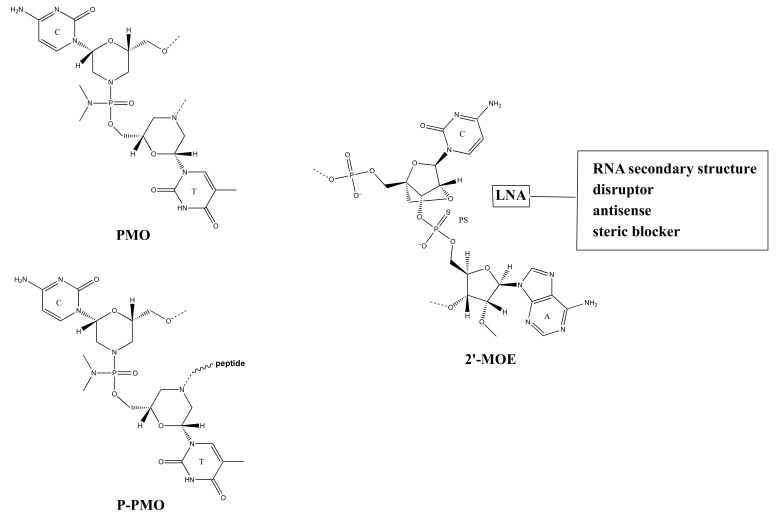
Structures of nucleic acid analog [PMO (morpholino), P-PMO (peptide-morpholino), LNA (locked nucleic acid)] moieties and modifications of the nucleic acid backbone [PS (phosphorothioate), 2′-MOE (2′-methyl O-ester)] employed in the antisense strategies against SARS-CoV-2.

The outbreak of SARS in 2003 (caused by SARS-CoV-1) and the COVID-19 pandemic sixteen years later showed the world our vulnerability to coronavirus infections [117]. Given that periodic outbreaks of similar pandemics could occur in the future, the scientific community and pharmaceutical companies should be prepared to fast-track the production of vaccines and antiviral oligonucleotides acting as RNA-targeting therapeutics in antisense strategies directed against coronaviruses. More specifically, oligonucleotides and NAAs can target the SARS-CoV-2 RNA genome and regulatory RNA sequences, disrupt RNA secondary structures or host protein/virus RNA complexes, and provoke steric blocks (Table 2). The first antisense oligonucleotides reported against SARS-CoV-1, whose genome is closely related to SARS-CoV-2, targeted the ORF1a gene and the transcription regulatory RNA sequence located in the 5′-UTR region of the positive-sense RNA genome of SARS-CoV-2 [118]. These antisense oligonucleotides (antisense morpholino oligomers and peptide-conjugated antisense morpholino oligomers [P-PMOs], Figure 5) were endowed with high antiviral activity in vitro, revealing the potential capacity of the antisense nucleic acid analogs (NAA) for antiviral treatment against SARS-CoV-1 and, potentially, SARS-CoV-2. This work acted as a proof of concept for the efficiency of RNA-targeting NAAs as an antiviral treatment in the context of human coronavirus infections [118].

Notably, the SARS-CoV-2 genome can be targeted at any step of its life cycle by antisense oligonucleotides that allow for targeting any of the conserved sequences of both positive and negative RNA. Regarding the structure of the SARS-CoV-2 genome, whose knowledge is necessary for developing specific antisense oligonucleotides, it consists of a single-stranded RNA of ca. 30,000 nucleotides capped at the 5′ end and endowed with a 3′ poly rA-tail, as well as two short UTR-sequences [118]. SARS-CoV-2 RNA genome encodes 14 ORFs, of which ORF1a and ORF1b, at the 5′ end, encode the replicase polyprotein comprising ~2/3 of the entire genome. In addition to these nonstructural proteins, the remaining genome contains nine small ORFs encoding structural proteins such as nucleocapsid (N), envelope (E), spike (S), membrane (M), and others with accessory roles. SARS-CoV-2 RNAs include both subgenomic and genomic entities, with the first RNA tracts being translated into structural proteins and others with accessory roles.

On the other hand, genomic RNA is involved in replicating viral RNA before its incorporation into virions. In principle, any sequence of SARS-CoV-2 RNA genome is a potential target of antisense oligonucleotides, but genomic RNAs and replication steps are especially recommended for antisense strategies as targeting subgenomic entities is associated with lower antiviral efficacies [118]. After infection, SARS-CoV-2 replicates its genome inside the human cell using enzymes for replication encoded by the coronavirus itself. More in detail, the 5′ cap, and 3′ poly rA tail modifications allow direct translation of the nonstructural proteins encoded by genes ORF1a and ORF1b. Afterward, the assembly of the replicase-transcriptase complex (RTC) occurs, leading to the replication of the RNA genome alongside the discontinuous subgenomic mRNA transcription, mediated by short AU-rich transcription regulatory sequences, whose resulting subgenomic RNAs are further translated into the structural proteins and the others with various accessory roles. Huston et al. [119] applied a novel long amplicon strategy to resolve the secondary RNA structure of the coronavirus genome in infected cells, which revealed an elaborate SARS-CoV-2 genome architecture that included well-folded RNA regions, some of which were unique while others were conserved across beta-coronaviruses. They developed antisense oligonucleotides containing LNA (Figure 5) moieties as steric blockers and tested them in targeting two putative RNA regions within the SARS-CoV-2 genome [119]. The two oligonucleotides contained three consecutive LNAs at their 5′ and 3′ termini, whereas unmodified consecutive nucleotides within the strands were limited to three to prevent RNase-dependent activation. These NAA-based antisense strategies significantly inhibited the growth of SARS-CoV-2, demonstrating that the two targeted secondary RNA structures are critical for the life cycle of the coronavirus and that these LNA-containing antisense oligonucleotides disclose potential as anti-COVID-19 therapeutics [119].

Further investigations used antisense oligonucleotides carrying 2′-*O*-methoxyethyl (2′-MOE, Figure 5) and phosphorothioate (PS, Figure 5) backbone modifications as steric blockers to disrupt interactions between the host RNA binding proteins and SARS-CoV-2 RNA thanks to their enhanced steric blocking activity [121]. Moreover, RNase H-dependent antisense oligonucleotides, carrying LNA tracts at both ends, were used to target the SARS-CoV-2 stem-loop 2 motif (s2m) in the 3′-UTR of the cytosolic positive-sense RNA strand [120]. In addition to the terminal LNA tracts, the antisense oligonucleotides carried long stretches of unlocked ribonucleotides that ensured RNase H recruitment and, therefore, viral RNA cleavage. Interestingly, as RNase H is responsible for the cleavage of the targeted RNA in the duplexes formed by RNA and the antisense oligonucleotide, this latter remains intact and free to bind to other RNAs, while the SARS-CoV-2 RNA is cleaved in a sequence-specific manner, which led to inhibition of the replication of SARS-CoV-2 in infected cells [120]. Overall, these anti-COVID-19 RNA-targeting strategies seem to be promising, especially in view of targeting conserved RNA tracts in different variants of SARS-CoV-2 and other human coronaviruses. However, these approaches still need further research supporting their translation into clinics.

### 3.3. Aptamers and G-Quadruplex Structures for the Detection of SARS-CoV-2

While most anti-COVID-19 strategies were designed to target essential proteins within the SARS-CoV-2 genome, targeting RNA structural elements is also of crucial importance especially using the class of oligonucleotide aptamers [122]. This family is composed of oligonucleotides of different nature that, similarly to antibodies, recognize specific three-dimensional structures acting as “chemical antibodies” [123]. Thanks to oligonucleotide aptamers’ high affinity and specificity for their targets, they offer unique chemical and biological characteristics rendering them particularly suitable for novel biomedical applications, including in vitro diagnosis, biomarkers discovery, in vivo imaging, and therapy [123]. The highly conserved RNA structure within the s2m motif of SARS-CoV-2 was targeted by high-affinity L-DNA aptamers [124] to evaluate their therapeutic and diagnostic potential. Optimized L-DNA aptamers were found to bind selectively to s2m with affinities in the nanomolar range and proved capable of discriminating between the monomeric s2m stem-loop and the homodimer duplex. The L-DNA mode of recognition is highly structure-specific, allowing to differentiate s2m RNAs from different but closely related human coronaviruses, such as SARS-CoV-1 and SARS-CoV-2, differing by only two ribonucleotides. In addition, L-DNA aptamers induce significant conformational changes in s2m RNA structure upon their molecular recognition, with a potential role in disrupting or preventing protein–s2m binding [124].

G-quadruplex (G4) DNA or RNA is a non-canonical secondary structure resulting from assembling one, two, or four guanine-rich nucleic acids strands into a quadruple helix structure stabilized by coordination with suitable monovalent cations [125]. G4 structure and function are determined by factors such as the number and polarity of nucleotide strands, the type of metal ions, as well as the structural properties of their binding targets [125]. Targeting of the G4-folded SARS-CoV-2 RNA genome by specific aptamers appears to be a promising alternative method for SARS-CoV-2 detection [126]. In addition to their importance as aptamer targets, G4-forming oligonucleotides can also be used to realize G4 aptamer-based biosensors to detect SARS-CoV-2 surface proteins. Indeed, G4-based biosensors represent a valuable alternative to antibody-based detection of SARS-CoV-2 and other pathogens [126].

The use of aptamers for targeting SARS-CoV-2 RNA genomic tracts in COVID-19 therapy and diagnostic approaches based on the recognition of virus proteins are promising strategies thanks to the intrinsic specificity guaranteed by the aptamer technology. Nevertheless, the utility of these approaches is still to be evaluated in prospective clinical and diagnostic studies.

## 4. Conclusions

The outbreak of the COVID-19 pandemic showed our vulnerability to coronavirus infections, and given that other pandemics could attack humanity after the current crisis, the scientific community, together with pharmaceutical companies, should be prepared to fast-track the production not only of vaccines, but also of nucleic acid-based antisense tools and aptamers acting as RNA-targeting therapeutics against coronaviruses. Overall, the herein summarized applications of nucleic acids, and especially RNAs and NAAs, in the context of the fight against SARS-CoV-2 demonstrate the feasibility of using nucleic acids for mass immunization when the urgency of counteracting the virus spread does not allow waiting for the development of long-established live attenuated and inactivated virus- and protein-based vaccines. Additionally, the above literature reports show the importance of targeting SARS-CoV-2 RNA using antisense oligonucleotides and aptamers, which has important implications in diagnosing and treating the infectious disease caused by SARS-CoV-2. Remarkably, the high affinity and selectivity of oligonucleotide antisense devices and aptamers, coupled with the intrinsic nuclease resistance of NAA that can be easily introduced in their structures, enable novel opportunities for generating new tools and probes for interrogating RNA function in SARS-CoV-2 and related coronaviruses.

## Figures and Tables

**Table 1:** Some of the most used nucleic acid-based vaccines in Western countries.

Vaccine Name	Carried Nucleic Acid	Developer	Confirmed Efficacy
ChAdOx1-S/AZD1222	DNA	AstraZeneca + University of Oxford	63.1%, based on a median follow-up of 80 days
Ad26.CoV2.S	DNA	Janssen Pharmaceuticals Johnson & Johnson	66.0%, 28 days post-vaccination
BNT162/Comirnaty	RNA	Pfizer/BioNTech + Fosun Pharma	95.0%, measured starting from seven days after the second dose
mRNA-1273	RNA	Moderna + National Institute of Allergy and Infectious Diseases (NIAID)	94.1%, measured starting from two weeks after the second dose

**Table 2:** Some of the most used nucleic acid analogs (NAA) and their main anti-SARS-CoV-2 applications.

NAA	Full Name	Properties	Reference
PMO/P-PMO	Morpholino/peptide-morpholino	Targeting RNA genome/regulatory sequences/very high nuclease stability	[118]
LNA	Locked nucleic acid	Disrupting RNA secondary structure/provoking steric blocks/very high nuclease stability	[119,120]
2′-MOE	2′-Methyl O-esters	Disrupting interactions between host proteins and SARS-CoV-2 RNA/provoking steric blocks/high nuclease stability	[121]
PS	Phosphorothioates	Disrupting interactions between host proteins and SARS-CoV-2 RNA/provoking steric blocks/high nuclease stability	[121]

## Data Availability

Not applicable.
